# High-Sensitivity Bio-Waste-Derived Triboelectric Sensors for Capturing Pathological Motor Features in Hemiplegia Rehabilitation

**DOI:** 10.3390/mi17040395

**Published:** 2026-03-25

**Authors:** Shengkun Li, Huizi Liu, Chunhui Du, Yanxia Che, Chengqun Chu, Xiaoyan Dai

**Affiliations:** 1 State Key Laboratory of Extreme Environment Optoelectronic Dynamic Measurement Technology and Instrument, North University of China, Taiyuan 030051, China; lishengkun@nuc.edu.cn (S.L.); 17696108825@163.com (H.L.); daixiaoyan@nuc.edu.cn (X.D.); 2 China Coal Technology and Engineering Group, Taiyuan Institute Co., Ltd., Taiyuan 030006, China; kzjdsdch@163.com; 3 Beijing Aurasky Electronics Co., Ltd., Beijing 100176, China; cheyanxiahao@126.com

**Keywords:** triboelectric nanogenerators, self-powered sensors, hemiplegia rehabilitation, pathological motor features, wearable electronics

## Abstract

Continuous monitoring of pathological motor features is vital for post-stroke rehabilitation but remains challenged by power reliance and low sensitivity of wearable sensors. Here, we develop a high-sensitivity, self-powered breathable nanogenerator (BN-TENG) utilizing fish-scale-derived biological hydroxyapatite/carbon (Bio-HAp/C) fillers within electrospun polyvinylidene fluoride (PVDF) nanofibers. The Bio-HAp/C enhances electron-trapping capability, while a high-resilience ethylene-vinyl acetate (EVA) spacer optimizes contact-separation dynamics. The BN-TENG achieves a superior sensitivity of 16.28 V·N^−1^ and remarkable stability over 10,000 cycles. By implementing a multi-node sensing strategy, the sensor successfully captures complex hemiplegic patterns, including compensatory shoulder hiking, distal muscle spasticity, and postural asymmetry. By resolving subtle micro-vibrations missed by traditional electronics, this work provides a sustainable, autonomous interface for characterizing pathological motor features and assessing rehabilitation progress in hemiplegic patients.

## 1. Introduction

Stroke is a premier etiology of protracted neurological disability, with millions of survivors grappling with hemiplegia annually [1,2,3]. The restoration of motor function is an arduous process governed by neuroplasticity [4,5,6]; however, this process is frequently compromised by maladaptive compensatory patterns and abnormal muscle synergies [7,8]. Specifically, movements such as shoulder hiking [9,10] and truncal postural asymmetry [11,12] not only impede the performance of activities of daily living (ADLs) but also risk reinforcing permanent pathological gait if not corrected early [13]. Consequently, there is an urgent need for continuous, multi-site biomechanical monitoring strategies to shift rehabilitation from subjective, intermittent clinical scales (e.g., Fugl-Meyer Assessment) toward objective, quantitative home-based monitoring [14,15].

While wearable electronics like inertial measurement units (IMUs) [16,17,18] and surface electromyography (sEMG) [19,20] have emerged as alternatives, they are inherently constrained by rigid form factors, “power anxiety” due to bulky batteries, and limited sensitivity to low-magnitude physiological tremors [21,22]. Specifically, IMUs often require complex filtering to compensate for drift during slow movements, while sEMG signals are highly susceptible to skin-electrode impedance fluctuations and electromagnetic interference. In contrast, triboelectric nanogenerators (TENGs) utilize the contact-separation mechanism to directly convert biomechanical deformations into robust voltage outputs. This mechanism allows for a high signal-to-noise ratio in capturing low-frequency pathological features—such as muscle spasticity and compensatory tremors—without the need for external biasing or frequent recharging. These bottlenecks severely hinder their seamless integration into long-term, skin-interfaced rehabilitation paradigms [23,24]. Self-powered triboelectric nanogenerators (TENGs) offer a compelling solution by transducing biomechanical energy into autonomous electrical signals [25]. Polyvinylidene fluoride (PVDF) is a primary candidate for such sensors due to its flexibility [26,27]. However, the low surface charge density of pristine PVDF restricts its ability to resolve subtle, high-frequency pathological motor features [28].

To enhance performance, modulating the dielectric properties of polymers via functional fillers has become a focal point [29,30]. Emerging trends emphasize biocompatible materials derived from natural biowaste, aligning with the “green electronics” movement [31,32,33,34,35]. Preliminary studies have explored the potential of upcycling biowaste as functional additives [36,37,38]. Among various biological precursors, fish scales represent an abundant and underutilized by-product of the seafood processing industry. Their hierarchical structure is primarily composed of collagen fibers mineralized with hydroxyapatite nanocrystals, providing a natural template for generating hybrid inorganic–carbon structures after thermal treatment. Such structures can introduce interfacial polarization centers and defect-mediated charge trapping sites when incorporated into polymer matrices. Nevertheless, the strategic utilization of such biomaterials to optimize electron-trapping efficiency and interfacial polarization remains underexplored, leaving a gap in developing high-sensitivity sensors capable of capturing complex, multi-regional pathological patterns [39,40,41].

In this work, we present a high-sensitivity, bio-waste-enhanced triboelectric sensor (BN-TENG) specifically engineered for the high-fidelity capture of hemiplegic motor signals. By incorporating fish-scale-derived biological hydroxyapatite/carbon (Bio-HAp/C) fillers into electrospun PVDF nanofibers, we significantly enhanced the charge-trapping capability and effective contact area. Supported by a high-resilience ethylene-vinyl acetate (EVA) spacer, the BN-TENG achieves a superior sensitivity of 16.28 V·N^−1^ and remarkable durability over 10,000 cycles. Crucially, by implementing a multi-node sensing strategy, this study demonstrates the sensor’s capacity to resolve intricate motor features, including compensatory shoulder elevation and limb spasticity, that are typically obscured in traditional electronics. This research provides a robust, sustainable interface for the digitized evaluation of motor dysfunction, establishing a foundation for next-generation self-powered pathological diagnostics and comprehensive whole-body rehabilitation monitoring.

## 2. Materials and Methods

### 2.1. Materials

Raw fish scales (FSs) are collected as biowaste from a local fishery market. Polyvinylidene fluoride (PVDF, powder) is purchased from Solvay (Shanghai, China). N,N-Dimethylformamide (DMF, 95% purity) is supplied by Shanghai Macklin Biochemical Co., Ltd. (Shanghai, China). Medical Non-woven Fabric is purchased from Alibaba Health Pharmacy Chain Co., Ltd. (Guangzhou, China). Adhesive EVA Spacer is custom-made Shenzhen Yichen Technology Co., Ltd. (Shenzhen, China). Silver conductive paste (Sigma-Aldrich) (Zhuhai, China) was employed as the electrode material.

### 2.2. Preparation of Functional Bio-HAp/C Filler

Waste fish scales are collected from a local seafood market and undergo a rigorous pretreatment process to ensure high purity. Fish scales are a typical biogenic composite composed primarily of hydroxyapatite minerals embedded within an organic collagen matrix, which makes them a suitable precursor for generating hybrid mineral–carbon structures after thermal conversion. The raw scales are first rinsed repeatedly to remove surface impurities and then immersed in deionized (DI) water for 48 h to ensure thorough hydration. Subsequently, a decalcification process is performed by stirring the scales in formic acid for 2 h using a magnetic stirrer, followed by multiple rinsing cycles with DI water until a neutral pH is achieved. After the cleaned scales are dried, a calcination process is executed at 450 °C for 4 h (with a heating rate of 10 °C/min in a tube furnace) to transform the biological precursor into a stable crystalline phase of Bio-HAp/C filler. During this process, partial carbonization of the organic matrix occurs while the mineral phase remains stable, forming heterogeneous mineral–carbon interfaces that can contribute to charge trapping within the triboelectric composite layer. Finally, the resulting material is mechanically ground into a fine powder, which serves as the functional Bio-HAp/C filler ready for subsequent electrospinning and composite film fabrication.

### 2.3. Preparation of PVDF/Bio-HAp/C Composite Membrane

The fabrication of the functional sensing layer initiates with the synthesis of a multi-component hybrid solution. Specifically, 2 g of PVDF powder and 0.024 g of the as-prepared Bio-HAp/C filler (corresponding to a concentration of approximately 1.2 wt%, optimized to balance the output performance and fiber morphology) are dispersed into a N,N-dimethylformamide (DMF) solvent system. To ensure a homogeneous distribution of the functional fillers within the polymer matrix, the mixture is subjected to continuous magnetic stirring at a controlled speed of 200 rpm and a constant temperature of 40 °C for a duration of 4 h. Upon achieving a stabilized precursor gel, the solution is transferred into a specialized syringe for the electrospinning process, where the synergistic effect between the Bio-HAp/C additives and the PVDF matrix facilitates the formation of a micro-nano fibrous network with enhanced piezoelectric and triboelectric characteristics.

### 2.4. Fabrication of the BN-TENG Sensing Unit

The architectural integration of the sensing unit follows a vertical assembly sequence. Initially, a silver electrode, featuring a 1 cm × 1 cm area and a 0.1 cm lead-out wire, is deposited onto a flexible PET substrate via screen printing. A customized spacer with identical dimensions is then securely bonded onto the electrode surface to define the internal hollow cavity. Subsequently, the as-prepared PVDF/Bio-HAp/C composite membrane is positioned atop the spacer, establishing a sandwich-like configuration that facilitates effective contact-separation dynamics. To ensure long-term stability and wearability for medical monitoring, the entire assembly is finalized by an encapsulation process using a biocompatible and breathable non-woven fabric, which provides structural protection while maintaining superior skin comfort.

### 2.5. Characterization and Measurement

The structural investigation of the as-prepared composite membranes is conducted via field emission scanning electron microscopy (SEM, FEI Nova NanoSEM 450, FEI, Hillsboro, OR, USA) to elucidate the surface topography and internal morphology. A single-zone tube furnace (OTF-1200X, Hefei Ke Jing Materials Technology Co., Ltd., Hefei, China) provides the controlled thermal environment required for the high-temperature calcination of biological precursors. Regarding the electrical characterization, the output signals of the BN-TENG are captured by a programmable electrometer (Keithley 6514, Keithley Instruments, Cleveland, OH, USA), while the corresponding potential waveforms are monitored using a multi-channel digital oscilloscope (Tektronix MDO34, Tektronix, Inc., Beaverton, OR, USA). Controlled mechanical excitation is applied through a customized vibration platform—integrating a signal generator, a power amplifier, and an electromagnetic shaker—to simulate various contact-separation dynamics with precise frequency and force regulation. Furthermore, all trials are performed following the acquisition of signed informed consent from each participant, ensuring ethical compliance throughout the experimental phase.

### 2.6. Data Acquisition and Rehabilitation Evaluation

To evaluate the practical utility of the BN-TENG in post-stroke rehabilitation, a sequential data acquisition protocol is implemented on a hemiplegic patient. The individual sensing unit is conformally attached to specific anatomical landmarks, including the upper trapezius, medial forearm, and lumbar region of the trunk, using medical-grade adhesive tape to ensure stable interface contact. During the monitoring phase, the sensor is connected to a high-impedance electrometer for the real-time capture of physiological electrical signals. Three standardized rehabilitation maneuvers are performed to identify abnormal motion patterns and assess the recovery status: arm elevation, fist clenching, and lateral trunk bending. By recording the voltage-time profiles at each site individually, the distinctive output characteristics (such as peak amplitude and signal periodicity) are analyzed to quantify compensatory motions and muscle spasticity, providing a preliminary metric for the patient’s functional restoration.

## 3. Results

### 3.1. Design Concept and Working Mechanism of the BN-TENG

**Figure 1:**
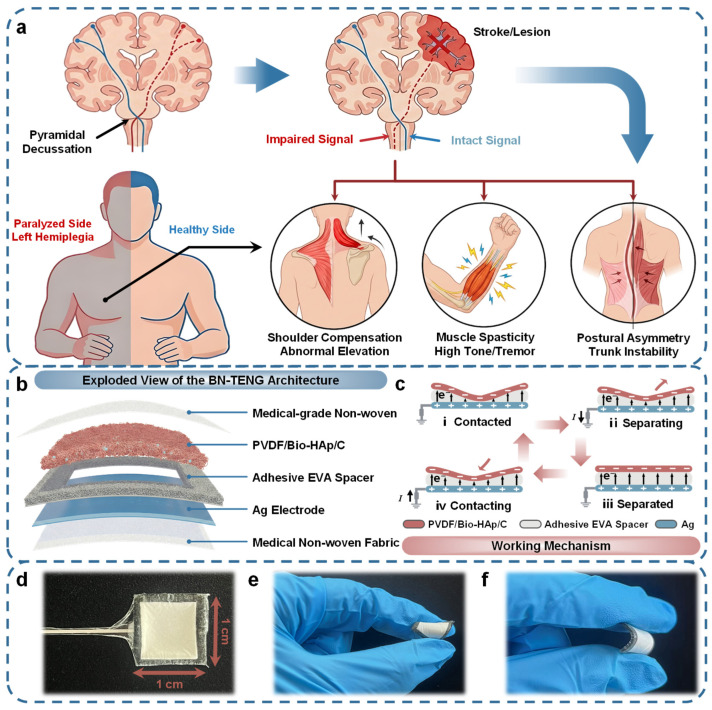
Design concept, structural architecture, and working principle of the BN-TENG. (**a**) Schematic illustration of the neurological mechanism of hemiplegia. (**b**) Exploded structural view of the BN-TENG. The red arrows represent one complete contact-separation cycle. (**c**) Schematic of the working mechanism based on the single-electrode contact-separation mode. (**d**) Optical photograph of the fabricated sensor unit with dimensions of 1 cm × 1 cm. (**e**,**f**) Photographs demonstrating the flexibility of the sensor under (**e**) twisting and (**f**) bending deformations.

The development of effective rehabilitation monitoring systems requires a profound understanding of the underlying neuropathology. As illustrated in Figure 1a, motor impairments in hemiplegia typically originate from neurological lesions within the motor cortex of the brain, such as those caused by stroke. Due to the anatomical mechanism of pyramidal decussation in the brainstem, neural pathways cross to the opposite side, meaning that a lesion in the right hemisphere inevitably results in paralysis or motor deficits on the left side of the body. This disruption of descending neural signals leads to a series of characteristic physiological anomalies that hinder daily life. These include compensatory shoulder elevation caused by the weakness of the deltoid muscle, upper-limb spasticity characterized by high muscle tone and tremors in the forearm, and postural asymmetry resulting from trunk muscle instability. Precise capturing of these pathological signals is critical for evaluating rehabilitation progress, which motivates the design of our sensing platform.

To address this need, we have developed a Breathable Nanofibrous Triboelectric Nanogenerator (BN-TENG) designed for conformal skin integration. Figure 1b presents the exploded view of the device’s innovative sandwich architecture. Unlike traditional sensors encapsulated in airtight elastomers (e.g., PDMS or Eco-flex), the BN-TENG employs medical-grade non-woven fabrics as the top encapsulation and bottom substrate, ensuring excellent air permeability and skin-friendly characteristics for wearable use. Such medical-grade nonwoven fabrics are widely used in medical dressings and wearable textiles due to their intrinsic breathability and soft contact interface with human skin. The core functional unit comprises a Ag Electrode and a specific negative triboelectric layer fabricated from PVDF/Bio-HAp/C nanofibrous membrane. Here, the Bio-HAp/C, derived from sustainable fish scale biowaste, acts as a functional filler to introduce deep-level charge traps. To maintain a stable air gap while preventing structural collapse, a customized Adhesive EVA Spacer with a porous sponge skeleton is inserted between the triboelectric layers. This porous EVA frame creates a stable cavity to facilitate sufficient contact-separation cycles while providing superior mechanical resilience against structural collapse compared to rigid spacers.

The energy harvesting and sensing capability of the BN-TENG operates on the vertical contact-separation mode, as depicted in Figure 1c. In the initial state, the triboelectric layers are separated by the EVA spacer. When external mechanical stimuli (such as muscle contraction or joint bending) compress the device (Figure 1c State i), the PVDF/Bio-HAp/C membrane contacts the Ag electrode, generating surface triboelectric charges due to the difference in electron affinity. As the pressure releases and the layers separate (Figure 1c State ii–iii), the potential difference drives free electrons to flow through the external circuit to balance the electrostatic field, generating an electrical signal. This process reverses upon re-contacting (Figure 1c State iv), producing an alternating current (AC) output that faithfully reflects the frequency and amplitude of the mechanical motion.

The fabricated sensor exhibits a compact footprint (1 cm × 1 cm) and a lightweight profile, as shown in Figure 1d. Crucially, for a wearable interface, mechanical compliance is paramount. The BN-TENG demonstrates exceptional flexibility and robustness; it can withstand complex deformations, including severe twisting (Figure 1e) and large-angle bending (Figure 1f), without structural delamination or fracture. This superior mechanical adaptability allows the sensor to conform seamlessly to curvilinear body surfaces—such as the neck, forearm, and waist—ensuring reliable signal acquisition even under the dynamic conditions of hemiplegic rehabilitation training.

### 3.2. Optimization and Characterization of the Bio-Doped Nanofibrous Negative Triboelectric Layer

**Figure 2:**
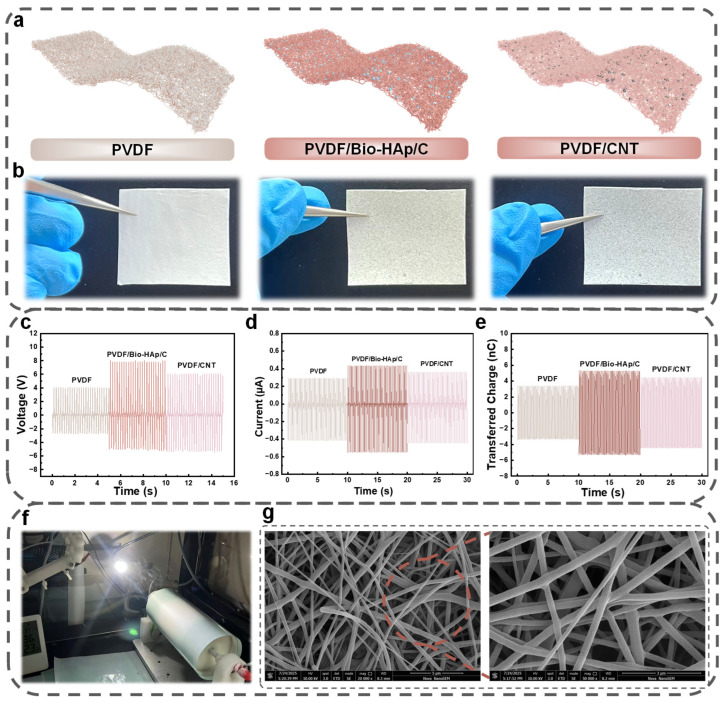
Material screening and characterization of the negative triboelectric layer. (**a**) Schematic illustrations of the pure PVDF, PVDF/Bio-HAp/C, and PVDF/CNT nanofibrous membranes. (**b**) Optical photographs showing the physical appearance of the three electrospun films. (**c**–**e**) Comparison of the electrical output performance among the three different materials, including (**c**) open-circuit voltage, (**d**) short-circuit current, and (**e**) transferred charge. (**f**) Optical photograph of the high-voltage electrospinning setup used for membrane fabrication. (**g**) SEM images of the optimized PVDF/Bio-HAp/C nanofibers at 5 µm and 3 µm scales, displaying the uniform distribution of functional particles within the fiber network.

The output performance of a TENG is intrinsically dictated by the surface charge density and electron-trapping capability of its friction materials. To identify the optimal constituent for the negative triboelectric layer, we systematically investigate three types of nanofibrous films: pure PVDF, PVDF doped with Bio-HAp/C, and PVDF doped with carbon nanotubes (CNTs). Figure 2a illustrates the schematic composition of these three candidate materials, highlighting the different functional fillers introduced into the polymer matrix.

The physical appearance and morphological characteristics of the fabricated films are presented in Figure 2b. The pure PVDF membrane appears pristine white with a relatively smooth macroscopic texture. In contrast, the PVDF/CNT film exhibits a greyish-black hue due to the intrinsic color of the carbon nanotubes. Notably, the PVDF/Bio-HAp/C film presents a distinctive yellowish tint. This functional filler is derived from a green fabrication strategy where discarded fish scales are collected and subjected to high-temperature calcination and fine grinding. This process effectively converts the biowaste into Bio-HAp/C powder, which is then uniformly dispersed into the spinning solution to impart enhanced triboelectric properties to the membrane. The incorporation of Bio-HAp/C fillers modulates the effective permittivity ($\varepsilon_{\mathrm{eff}}$) of the PVDF nanofibrous membrane, which directly influences the capacitive charge storage capacity. According to the Maxwell-Wagner effect at the filler-matrix interface, the polarization charge density (P) can be described as:

(1) $$P=\varepsilon_{0}\left(\varepsilon_{r}-1\right)E$$

where $\varepsilon_{r}$ is the relative permittivity of the Bio-doped composite and E is the internal electric field. The high-surface-area Bio-HAp/C particles act as deep-level trapping centers, effectively pinning the injected electrons and preventing their recombination, thereby maintaining a high surface charge density during dynamic rehabilitation movements.

To quantitatively evaluate the contribution of these fillers to energy harvesting, we assemble TENG devices using these three membranes as the negative triboelectric layer and compare their electrical output under identical mechanical excitation. As plotted in Figure 2c–e, the pure PVDF device yields the lowest baseline performance, with an open-circuit voltage (V_OC_) of 6.72 V, a short-circuit current (I_SC_) of 0.71 µA, and a transferred charge (Q_tr_) of 6.79 nC. The introduction of CNTs improves the output to 11.38 V, 0.81 µA, and 8.96 nC, likely due to the increased dielectric constant and conductivity of the network. However, the PVDF/Bio-HAp/C membrane exhibits the most superior performance among the three, achieving a V_OC_ of 12.8 V, an I_SC_ of 0.99 µA, and a Q_tr_ of 10.69 nC. This significant enhancement can be attributed to the Bio-HAp/C particles acting as dense charge-trapping sites within the polymer matrix, which effectively capture electrons and minimize surface charge dissipation during contact-separation cycles. The charge-trapping mechanism and the resulting temporal stability of the surface charge density (σ) can be further characterized by the decay model:

(2) $$\sigma (t)=\sigma_{0}\cdot exp\left(-\frac{t}{\tau }\right)$$

where $\sigma_{0}$ is the initial surface charge density immediately after contact electrification, and τ is the characteristic decay time constant. The incorporation of Bio-HAp/C significantly increases τ by introducing deep-level traps, which ensures that the captured electrons remain localized on the membrane surface for an extended duration, thereby sustaining high electrical output during repetitive rehabilitation exercises.

The fabrication of the functional triboelectric membranes is anchored by a precisely controlled high-voltage electrospinning process (Figure 2f), which serves as the structural foundation for achieving a high-performance active layer. As shown in the Scanning Electron Microscopy (SEM) images (Figure 2g), this technique yields a non-woven network characterized by randomly oriented nanofibers with a high degree of intrinsic porosity. This hierarchical porous architecture is double-functional: it ensures excellent air permeability for wearable comfort and significantly expands the effective specific surface area for charge induction [42,43]. The interconnected pore channels within the nanofiber network allow air and moisture to diffuse through the membrane, which helps reduce the occlusive effect commonly observed in dense polymer films used in conventional triboelectric devices. Crucially, high-magnification observation (inset of Figure 2g) reveals that the Bio-HAp/C particles are uniformly encapsulated within or anchored onto the PVDF nanofiber matrix without observable agglomeration. This homogeneous distribution is vital, as the embedded Bio-HAp/C fillers act as discrete polarization centers and charge-trapping sites. The resulting rough, composite topography not only maximizes the geometric contact area during the contact-separation cycles but also increases the density of functional sites available for contact electrification. Consequently, the synergistic effect between the nanofibrous framework and the Bio-HAp/C fillers establishes the PVDF/Bio-HAp/C membrane as a superior negative triboelectric layer.

It should be noted that the enhanced electrical output of the PVDF/Bio-HAp/C membrane cannot be attributed solely to a single mechanism. In triboelectric nanogenerators, the overall performance is typically governed by the synergistic interaction between dielectric polarization, surface morphology, and effective contact area. In the present system, the incorporation of Bio-HAp/C fillers not only introduces heterogeneous interfaces that can serve as potential charge-trapping sites but also modifies the effective dielectric environment of the composite membrane. Meanwhile, the electrospun nanofibrous structure inherently increases surface roughness and expands the geometric contact area during contact–separation cycles. The combined influence of these factors contributes to the improved charge generation and retention behavior observed in the BN-TENG.

### 3.3. Optimization of Spacer Architecture and Flexibility Characterization

**Figure 3:**
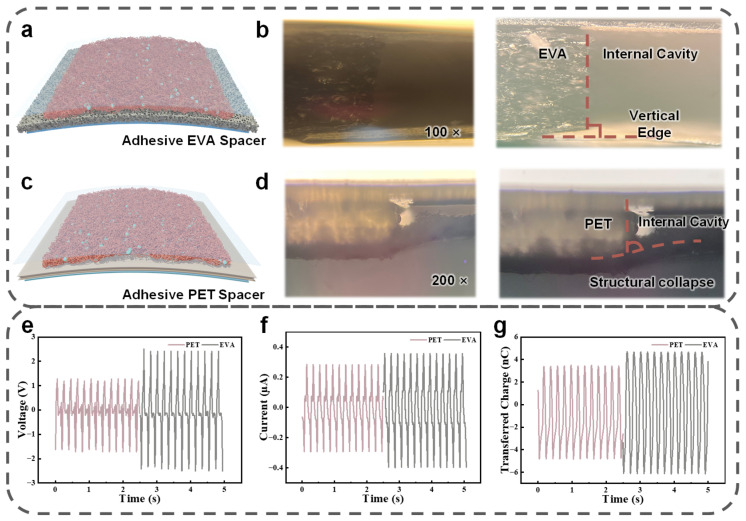
Selection of spacer materials and structural stability analysis. (**a**) Structural schematic of the BN-TENG with an adhesive EVA spacer. (**b**) Optical microscopy image of the EVA boundary interface, showing a stable vertical edge. (**c**) Structural schematic of the sensor utilizing a PET spacer. (**d**) Optical microscopy image of the PET interface, revealing significant structural collapse. (**e**–**g**) Comparative electrical output performance including (**e**) V_OC_, (**f**) I_SC_, and (**g**) Q_tr_ between EVA and PET-supported sensors.

The selection of an appropriate spacer material is fundamental to maintaining a stable dielectric gap and ensuring efficient contact-separation cycles within wearable triboelectric nanogenerators. We systematically investigate the mechanical and electrical characteristics of two candidate materials: a flexible adhesive ethylene-vinyl acetate (EVA) spacer and a conventional rigid polyethylene terephthalate (PET) spacer. Figure 3a,c present the structural schematics of the BN-TENG utilizing these two spacer configurations. To meticulously examine the structural integrity at the interface boundary, we perform optical microscopy at the spacer-film junctions. Under 100 times total magnification, the 1 mm thick EVA spacer demonstrates a robust vertical edge profile (Figure 3b). This stability stems from the unique porous skeleton of the EVA copolymer, which provides sufficient mechanical modulus to resist sagging while offering superior elasticity. In contrast, the 200 µm PET spacer, observed under 200 times total magnification, exhibits significant structural collapse at the junction (Figure 3d). This vulnerability is primarily attributed to the rigid nature of PET, which lacks the intrinsic resilience required to sustain a uniform air gap under the subtle pre-strains often encountered in flexible architectures.

The impact of these structural differences on electrical performance is quantified under identical periodic mechanical excitation. As plotted in Figure 3e–g, the BN-TENG with the EVA spacer achieves a peak V_OC_ of 4.93 V, an I_SC_ of 0.75 µA, and a Q_tr_ of 10.78 nC. These outputs significantly outperform the PET-based device, which yields a V_OC_ of only 2.99 V, an I_SC_ of 0.58 µA, and a Q_tr_ of 8.34 nC. This disparity can be analyzed through the equivalent capacitance model of triboelectric generators. The total capacitance (C) of the device is inversely proportional to the separation distance (d). In the EVA-supported structure, the high resilience of the sponge ensures a maximized and consistent d_max_ during the separation phase, leading to a larger variation in capacitance (ΔC) and a higher potential difference. Conversely, the structural collapse in the PET device reduces the effective separation stroke, thereby suppressing the electrostatic induction efficiency.

**Figure 4:**
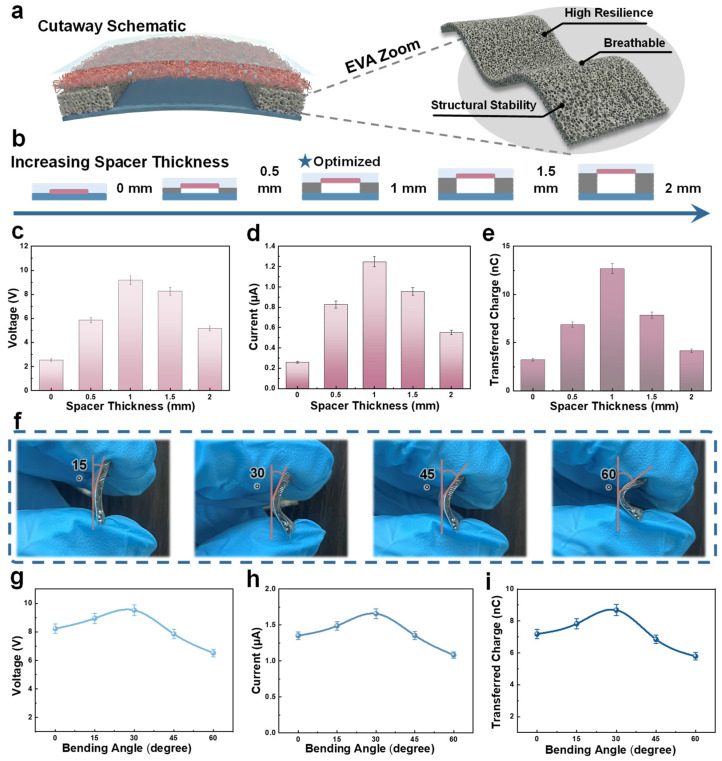
Structural parameter determination and flexibility characterization of the BN-TENG. (**a**) Cross-sectional schematic of the internal induction chamber and an enlarged view of the high-resilience, breathable EVA sponge skeleton. (**b**) Schematic illustration of the sensors fabricated with varying spacer thicknesses (0–2 mm). (**c**–**e**) Electrical output as a function of spacer thickness: (**c**) V_OC_, (**d**) I_SC_, and (**e**) Q_tr_. (**f**) Optical photographs of the sensing unit at different bending angles (θ). (**g**–**i**) Dynamic response of the sensor under varying bending angles: (**g**) V_OC_, (**h**) I_SC_, and (**i**) Q_tr_, featuring spline-fitted curves to highlight the performance peak at 30°.

Building upon the superior performance of the EVA architecture, we further explore the optimization of the internal cavity. Figure 4a provides a cross-sectional perspective of the optimized cavity, alongside a magnified view of the EVA sponge skeleton. This porous matrix not only ensures structural stability and high mechanical resilience but also facilitates a breathable pathway essential for long-term skin-interfaced applications. Meanwhile, the interconnected porous skeleton maintains mechanical resilience while avoiding the formation of an occlusive structure that could otherwise compromise wearable comfort during prolonged operation. To identify the optimal induction distance, we fabricate sensors with five different spacer thicknesses ranging from 0 mm to 2 mm (Figure 4b). As shown in Figure 4c–e, the electrical output exhibits a clear non-monotonic trend, peaking at a thickness of 1 mm with a V_OC_ of 9.16 V and Q_tr_ of 12.68 nC. Theoretically, within a specific range, increasing the air gap distance enhances the output voltage according to the relationship:

(3) $$V_{OC}=\frac{\sigma \cdot d(t)}{\varepsilon_{0}}$$

where σ is the surface charge density and d(t) is the instantaneous separation distance. However, as the thickness increases beyond 1 mm, the performance attenuates. This is primarily because excessive gap height increases the internal impedance and reduces the effective electric field coupling between the triboelectric charges on the negative triboelectric layer and the induction electrode.

Finally, to validate the sensor’s adaptability for monitoring diverse anatomical sites such as knuckles and wrists, we evaluate its performance under various bending states. Figure 4f captures the optical images of the sensing unit at bending angles (θ) from 0° to 60°. The corresponding output curves in Figure 4g–i are processed using a fitting algorithm, which effectively smooths experimental fluctuations to clearly reveal the underlying performance trend. The results demonstrate that the BN-TENG maintains robust output across a wide angular range, with a maximum performance observed at 30° (approx 9.52 V). This slight boost at moderate angles is attributed to the “pre-stress effect,” where the initial curvature tightens the contact interface and ensures more uniform contact-separation cycles. Although the output begins to decrease at extreme angles like 60° due to the partial occlusion of the internal cavity, the sensor retains sufficient signal integrity to capture subtle skin deformations and joint movements, making it an ideal candidate for precise, anatomy-integrated gesture recognition.

### 3.4. Electrical Performance and Sensing Characteristics of the BN-TENG

**Figure 5:**
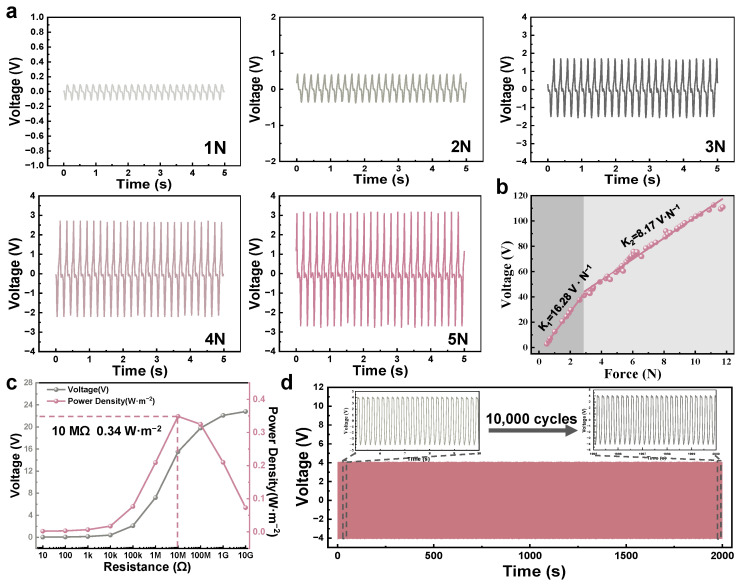
Electrical output and sensing performance characterization of the BN-TENG sensing unit. (**a**) Real-time output voltage response of a single BN-TENG unit under different impact forces (1 N to 5 N) at a fixed frequency. (**b**) Sensitivity analysis of the BN-TENG showing the linear relationship between output voltage and applied force, where the pink dots and lines represent the experimental data points and the corresponding linear fits, respectively. (**c**) Output voltage and corresponding power density of the BN-TENG as a function of external load resistance, showing a peak power density of 0.34 W·m^2^ at 10 MΩ. (**d**) Long-term stability test over 10,000 continuous cycles; insets show the detailed voltage waveforms at the beginning and the end of the test, highlighting the excellent signal consistency and mechanical durability.

To comprehensively evaluate the potential of the BN-TENG as a high-performance biomechanical sensor for clinical rehabilitation monitoring, we have conducted a systematic characterization of its dynamic electrical response. The fundamental sensing capability is first assessed by investigating the relationship between external mechanical stimuli and electrical output. As demonstrated in Figure 5a, the output voltage waveforms recorded at a constant frequency exhibit a clear and monotonic increase as the applied impact force rises from 1 N to 5 N. This positive correlation is fundamentally rooted in the effective contact mechanics at the solid–solid interface. Under higher mechanical loads, the negative triboelectric layer undergoes more significant macroscopic deformation, which promotes a more intimate and extensive contact with the silver electrode. This increase in the effective contact area allows for a higher density of surface charge transfer during the electrification process, thereby elevating the induced potential difference.

The quantitative relationship between the peak voltage and the applied force is further elucidated through linear fitting in Figure 5b. To quantitatively evaluate the sensing performance, the pressure sensitivity (S) of the BN-TENG is defined as the slope of the output voltage versus the applied mechanical force, which can be expressed as:

(4) $$S=\frac{\partial V}{\partial F}$$

where ∂V represents the change in peak-to-peak output voltage and ∂F is the variation in the applied impact force. Following this definition, the BN-TENG demonstrates an exceptional sensitivity of 16.28 V·N^−1^ in the lower force regime (under 3 N), which subsequently transitions to 8.17 V·N^−1^ for forces exceeding 3 N. This high sensitivity in the low-pressure range is particularly advantageous for capturing subtle physiological signals, such as the high-frequency muscle tremors associated with spasticity in a hemiplegic arm or the slight compensatory shrugging of the neck. The sustained sensitivity at higher forces further ensures that the sensor can accommodate a wide dynamic range, effectively monitoring larger-scale movements like postural shifts in the waist without signal saturation.

To evaluate the energy conversion efficiency and identify the internal impedance of the system, the BN-TENG is tested under a wide spectrum of external load resistances (R_L_) ranging from 10 Ω to 10 GΩ. As shown in Figure 5c, the output voltage progressively increases with R_L_ while the current density simultaneously declines, which is characteristic of a high-impedance power source. The instantaneous power density ($P_{d}$) delivered to the external load can be quantified by the following equation:

(5) $$P_{d}=\frac{V_{peak}^{2}}{R_{L}\cdot A}$$

where $V_{peak}$ is the peak output voltage across the load and A represents the effective contact area of the BN-TENG. Based on this relationship, the peak power density is calculated to be 0.34 W·m^−2^ at an optimal matching impedance of 10 MΩ. This impedance value reflects the capacitive nature of the device, where the maximum power transfer occurs when the external load matches the internal reactance of the TENG.

Finally, the operational durability and signal consistency of the BN-TENG are verified through long-term continuous excitation. Figure 5d illustrates the stability test results over 10,000 cycles under a repetitive 5 Hz impact. The output voltage remains remarkably stable throughout the duration of the test, with the magnified insets in Figure 5d showing nearly identical waveforms at the initial stage (5–10 s) and the final stage (1995–2000 s). This exceptional robustness confirms that the integrated structure of the PVDF/Bio-HAp/C membrane and the adhesive EVA spacer can withstand prolonged mechanical stress without structural fatigue or charge dissipation. To further verify the reliability and device-to-device reproducibility of the proposed sensor, we conducted comparative tests on five independent BN-TENG samples fabricated using the same Bio-HAp/C-doped PVDF nanofibers. The results exhibited that the output voltage deviations among these devices remained below 4.8% under a constant force of 5 N, indicating that the standardized electrospinning and assembly processes ensure high consistency in sensing performance. Collectively, these electrical characterizations demonstrate that the BN-TENG possesses the necessary sensitivity, stability, and signal-to-noise ratio to accurately detect and distinguish abnormal biomechanical signals in clinical rehabilitation scenarios.

### 3.5. Real-Time Monitoring and Feature Extraction of Hemiplegic Motor Impairments

**Figure 6:**
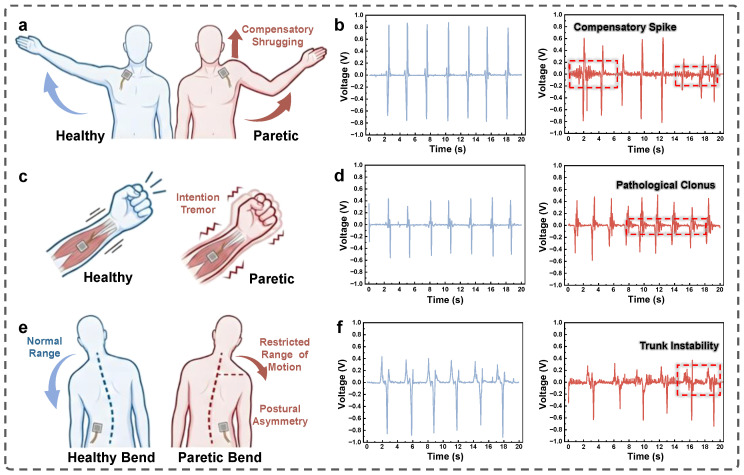
Real-time monitoring of hemiplegia rehabilitation metrics using the BN-TENG. (**a**) Schematic illustration of the sensor placement on the upper trapezius to monitor shoulder movements during arm abduction for both healthy and hemiplegic subjects. (**b**) Comparison of real-time output voltage signals recorded from the shoulder region, displaying the distinct signal patterns between normal lifting and compensatory shrugging. (**c**) Schematic illustration of the sensor attached to the inner forearm to detect muscle activity during a fist-clenching motion. (**d**) Real-time voltage response curves contrasting the signal characteristics of a healthy arm with those of an arm exhibiting tremors. (**e**) Schematic illustration of the sensor deployment on the lower back to monitor waist movements during lateral bending. (**f**) Output voltage signals recorded from the waist, comparing the waveform amplitudes and patterns between the healthy side and the paretic side. In all graphs, the blue and red lines represent the signals acquired from the healthy and paretic sides, respectively.

To validate the translational potential of the BN-TENG in clinical rehabilitation, we have deployed the sensors to monitor specific motor impairments in patients with hemiplegia. It is essential to note that the clinical utility of the BN-TENG lies in its ability to provide a direct, high-fidelity mapping of biomechanical stress into electrical signatures. Rather than relying on intermittent qualitative assessments, the distinct variations in voltage amplitude and frequency within the captured waveforms serve as intuitive indicators of motor coordination and muscle activation intensity. Upper limb motor recovery is frequently hindered by maladaptive compensatory movements, particularly in the shoulder complex. As illustrated in Figure 6a, patients with deltoid weakness often exhibit abnormal elevation of the scapula using the upper trapezius muscle when attempting to abduct the arm. This pathological synergy pattern not only restricts the functional range of motion but can also lead to long-term shoulder pain and recovery stagnation. We position the sensing unit on the upper trapezius of both a healthy volunteer and a volunteer with left-sided hemiplegia to monitor the signal evolution during arm-raising tasks. The corresponding real-time voltage response in Figure 6b reveals a distinct disparity. The healthy side maintains a relatively low and stable baseline signal, reflecting normal deltoid-driven movement. Conversely, the paretic side exhibits sharp, high-amplitude voltage spikes immediately prior to and during the arm elevation. This phenomenon indicates that the sensor successfully captures the intense, involuntary contraction of the trapezius muscle, providing a quantifiable indicator of compensatory shrugging. Such real-time detection of abnormal pressure surges is essential for delivering immediate biofeedback to patients, thereby discouraging incorrect motor patterns.

Distal muscle spasticity represents another pervasive challenge in stroke survivors, typically manifesting as hypertonia in the flexor muscles of the forearm. This condition often results in the involuntary curling of fingers and the wrist, known as flexor synergy. To evaluate the sensor’s capacity for spasticity quantification, we monitor the forearm flexor group during a fist-clenching motion, as shown in Figure 6c. The signal comparison in Figure 6d highlights the sensor’s exceptional dynamic response. On the healthy side, the voluntary contraction generates a smooth, low-frequency waveform characteristic of controlled muscle engagement. In contrast, the signal from the paretic side is superimposed with chaotic, high-frequency voltage fluctuations. These dense oscillatory signals correspond to pathological tremors and clonus, which typically occur in the frequency range of 5 to 10 Hz. The ability of the device to capture these micro-vibrations is directly attributed to the high surface charge density of the negative triboelectric layer, which ensures a high signal-to-noise ratio even under subtle skin deformations. This data suggests that the BN-TENG can serve as a digitized alternative to subjective manual spasticity assessments.

Foundationally, trunk control is critical for maintaining balance and preventing secondary complications such as pressure ulcers in wheelchair-bound patients. Hemiplegic individuals often suffer from core instability, leading to an involuntary list toward the unaffected or affected side and increasing the risk of falls. We investigate this postural asymmetry by placing sensors bilaterally on the waist and instructing the subjects to perform lateral bending motions (Figure 6e). The recorded signals in Figure 6f demonstrate a clear symmetry in the healthy subject, with balanced signal amplitudes during left and right bends. However, the patient data reveals a significant signal differential, where the voltage amplitude on the paretic side is markedly attenuated and irregular during the bending cycle. This reduced signal amplitude correlates with the restricted range of motion and muscular weakness inherent to the paretic core muscles. By detecting such signal asymmetries, this multi-node sensing strategy provides a reliable metric for evaluating core stability and postural alignment, offering a comprehensive solution for whole-body rehabilitation monitoring. It should be noted that this study serves as a preliminary technical validation of the BN-TENG platform. Although the current human-subject experiments involve a limited number of participants, the results clearly demonstrate the sensor’s superior ability to capture high-fidelity biomechanical signals.

## 4. Discussion

The development of the BN-TENG sensor unit represents a significant advancement in the integration of sustainable biowaste materials with high-performance wearable electronics. By synergistically combining electrospun PVDF nanofibers with Bio-HAp/C fillers derived from upcycled fish scales, we achieved a significant enhancement in surface charge density and electron-trapping capability. This material optimization, complemented by a structural strategy utilizing a high-resilience EVA spacer, ensures both exceptional sensitivity 16.28 V∙N^−1^ and mechanical durability over 10,000 cycles. Our results demonstrate that the sensor unit can effectively resolve subtle physiological signals, such as high-frequency tremors and compensatory muscle contractions, which are often missed by traditional rigid sensors. Unlike conventional active electronics that suffer from “power anxiety,” the BN-TENG operates as an autonomous interface, converting biomechanical energy directly into high-fidelity diagnostic signals. While this preliminary study involves a limited participant group, it successfully validates the technical feasibility of using Bio-HAp/C-based sensors for capturing specific motor signatures. This work provides a green, low-cost solution for the digital monitoring of hemiplegia-related motor features and establishes a robust foundation for high-sensitivity, self-powered rehabilitation sensing.

## Data Availability

The original contributions presented in this study are included in the article. Further inquiries can be directed to the corresponding author.
